# Superoxide Dismutase 3-Transduced Mesenchymal Stem Cells Preserve Epithelial Tight Junction Barrier in Murine Colitis and Attenuate Inflammatory Damage in Epithelial Organoids

**DOI:** 10.3390/ijms22126431

**Published:** 2021-06-16

**Authors:** Lee-Jung Tak, Hae-Young Kim, Won-Kook Ham, Gaurav Agrahari, Yoojin Seo, Ji Won Yang, Eun-Joo An, Chul Hwan Bang, Min Jung Lee, Hyung-Sik Kim, Tae-Yoon Kim

**Affiliations:** 1 Department of Dermatology, Seoul St. Mary’s Hospital, College of Medicine, The Catholic University of Korea, Seoul 06591, Korea; flwjd6744@naver.com (L.-J.T.); ho-jh1122@hanmail.net (H.-Y.K.); ham2326@naver.com (W.-K.H.); agra.gaurav06@gmail.com (G.A.); enjooida@nate.com (E.-J.A.); mrbangga@gmail.com (C.H.B.); princessmin@hanmail.net (M.J.L.); 2 Department of Oral Biochemistry, School of Dentistry, Pusan National University, Yangsan 50612, Korea; amaicat24@naver.com (Y.S.); midnightnyou@naver.com (J.W.Y.); 3 Dental and Life Science Institute, Pusan National University, Yangsan 50612, Korea

**Keywords:** superoxide dismutase 3, mesenchymal stem cell, inflammatory bowel disease, epithelial tight junction, immunomodulation, intestinal epithelial organoid

## Abstract

Superoxide dismutase 3 (SOD3), also known as extracellular superoxide dismutase, is an enzyme that scavenges reactive oxygen species (ROS). It has been reported that SOD3 exerts anti-inflammatory abilities in several immune disorders. However, the effect of SOD3 and the underlying mechanism in inflammatory bowel disease (IBD) have not been uncovered. Therefore, in the present study, we investigated whether SOD3 can protect intestinal cells or organoids from inflammation-mediated epithelial damage. Cells or mice were treated with SOD3 protein or SOD3-transduced mesenchymal stem cells (MSCs). Caco-2 cells or intestinal organoids stimulated with pro-inflammatory cytokines were used to evaluate the protective effect of SOD3 on epithelial junctional integrity. Dextran sulfate sodium (DSS)-induced colitis mice received SOD3 or SOD3-transduced MSCs (SOD3-MSCs), and were assessed for severity of disease and junctional protein expression. The activation of the mitogen-activated protein kinase (MAPK) pathway and elevated expression of cytokine-encoding genes decreased in TNF-α-treated Caco-2 cells or DSS-induced colitis mice when treated with SOD3 or SOD3-MSCs. Moreover, the SOD3 supply preserved the expression of tight junction (ZO-1, occludin) or adherence junction (E-cadherin) proteins when inflammation was induced. SOD3 also exerted a protective effect against cytokine- or ROS-mediated damage to intestinal organoids. These results indicate that SOD3 can effectively alleviate enteritis symptoms by maintaining the integrity of epithelial junctions and regulating inflammatory- and oxidative stress.

## 1. Introduction

Inflammatory bowel disease (IBD), including ulcerative colitis (UC) and Crohn’s disease (CD), is a complex multifactorial disease that affects the gastrointestinal tract with chronic and excessive inflammation. It commonly refers to the two chronic conditions that involve inflammation of the intestine [1]. The etiology and pathogenesis of these disorders have not yet been fully defined. Recent studies have indicated that a complex interplay of genetic, environmental, and microbial factors results in sustained aberrant innate immunity, followed by dysregulated adaptive immune responses [1]. Mitogen-activated protein kinases (MAPK) are Ser/Thr protein kinases that respond to extracellular stimuli such as growth factors and stress, and regulate various cellular activities including gene expression, mitosis, differentiation, and apoptosis. The MAPK signaling pathway is reported to modulate the expression of several tight junction proteins, thereby altering molecular composition within tight junction complexes [2]. Furthermore, the activation of MAPK signaling leads to elevated production and secretion of proinflammatory cytokines, such as interleukin (IL)-1β, IL-6, IL-8, and tumor necrosis factor (TNF)-α in IBD [3]. Regulation of oxidative stress is important in the maintenance of intestinal homeostasis. In homeostatic state, intestinal ROS have bactericidal effects, contributing to the intestinal defense function. However, oxidative stress derived from excessive ROS production can cause lipid peroxidation, intestinal mucosal barrier damage, bacterial translocation, and an inflammatory response. In UC, one type of IBD, oxidative stress has been shown to play a critical role in its pathogenesis and malignant progression to colorectal cancer (CRC) [4]. Moreover, evidence of ROS-induced epithelial damage during IBD progression have been suggested [5].

Therefore, we hypothesized that a potent antioxidant enzyme might contribute to the resolution of inflammation of IBD, thereby exerting therapeutic effects. SOD3 is an extracellular type of the superoxide dismutase family [6]. Several studies have proven that SOD3 can protect tissues from oxidative stress [7,8]. SOD3 has been demonstrated to attenuate excessive inflammation and modulate cytokine responses in chronic inflammation [9,10,11]. Moreover, previous studies by Mañes group have demonstrated that SOD3 is involved in the regulation of cell barrier function [12,13]. In our previous study, we proposed that direct administration of SOD3 protein, as well as MSC-delivered SOD3 can exert efficient therapeutic efficacy against allergic dermatitis, allergic airway inflammation, and psoriasis-like skin inflammation [14,15,16]. Interestingly, according to previous studies, the expression level of SOD3 was decreased in IBD-affected tissues of both animal models and patients [17,18,19]. Given that the immunoregulatory function of MSCs against several inflammatory diseases was augmented by the introduction of SOD3 into cells, in the present study, we sought to verify the combined therapeutic efficacy of SOD3 and MSCs in IBD.

Tumor necrosis factor-α (TNF-α) is a critical proinflammatory mediator in both acute and chronic stages of IBD [20]. TNF-α is one of the potent activators of the MAPK signaling pathway and plays a pivotal role in proinflammatory cytokine production in IBD [21]. More importantly, TNF-α-induced increase in intestinal epithelial tight junction permeability has been proposed as one of the pathophysiologic mechanisms contributing to intestinal inflammation [22]. Therefore, in this study, we assessed the anti-inflammatory abilities of SOD3 against inflammatory damage induced by TNF-α treatment in Caco-2 cells and further analyzed the alteration in the expression of tight junction proteins. In vivo experiments using a DSS-induced murine model of colitis were also conducted to evaluate the therapeutic efficacy of SOD3 or SOD3-MSCs. Moreover, to separately investigate the influence of SOD3 on homeostasis and regeneration of intestinal epithelium, intestinal organoids were utilized.

## 2. Results

### 2.1. SOD3 Regulates the Inflammatory Responses of TNF-α Treated Caco-2 Cells

**Figure 1:**
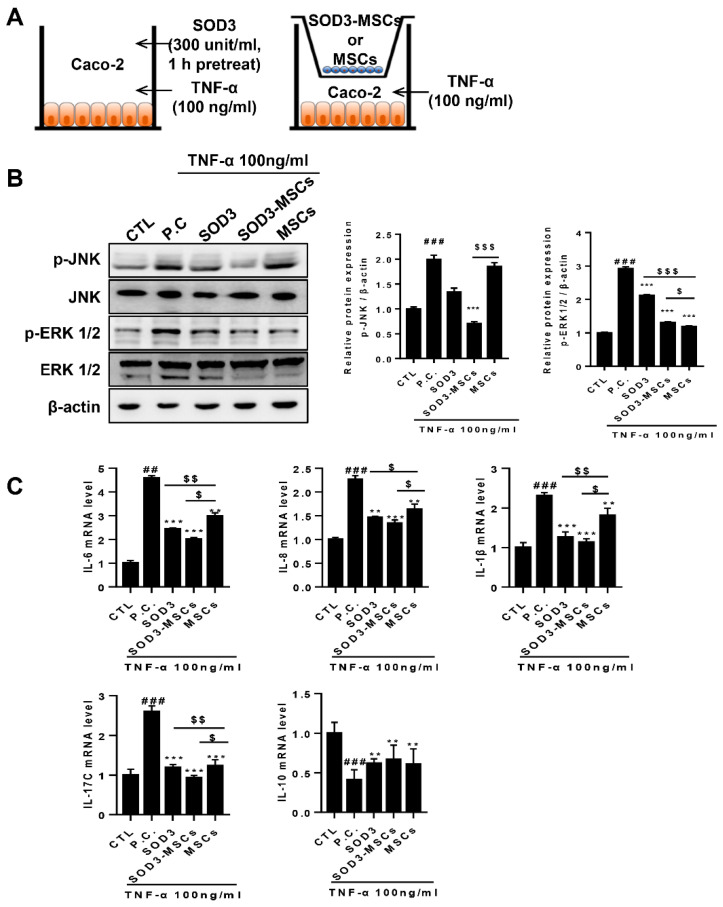
SOD3 and SOD3-MSCs inhibit the MAPK activation and inflammatory responses in response to TNF-α treatment. SOD3 (300 unit/mL) pretreatment or co-culture with SOD3-MSCs or MSCs were conducted to determine inflammatory activation of Caco-2 cells treated with TNF-α (100 ng/mL). (**A**) Schematic diagram of in vitro experiments. After harvesting Caco-2 cells, RNA or protein was extracted for further analysis. (**B**) The expression of p-JNK, JNK, p-ERK1/2, and ERK1/2 was determined by western blotting. (**C**) The expression of pro- (IL-6, IL-8, IL-1β, IL-1 and IL-17C) or anti-inflammatory (IL-10) cytokines in mRNA level was determined by quantitative PCR. CTL: untreated control; P.C: TNF-α-treated positive control, ## *p* < 0.01, ### *p* < 0.001 (CTL vs. P.C), ** *p* < 0.01, *** *p* < 0.001 (P.C vs. SOD3, SOD3-MSC and MSCs), $ *p* < 0.05, $$ *p* < 0.01, $$$ *p* < 0.001 (SOD3, SOD3-MSCs vs. MSCs). Results are shown as mean ± SD. Results show one representative experiment of at least three independent experiments.

**Figure 2:**
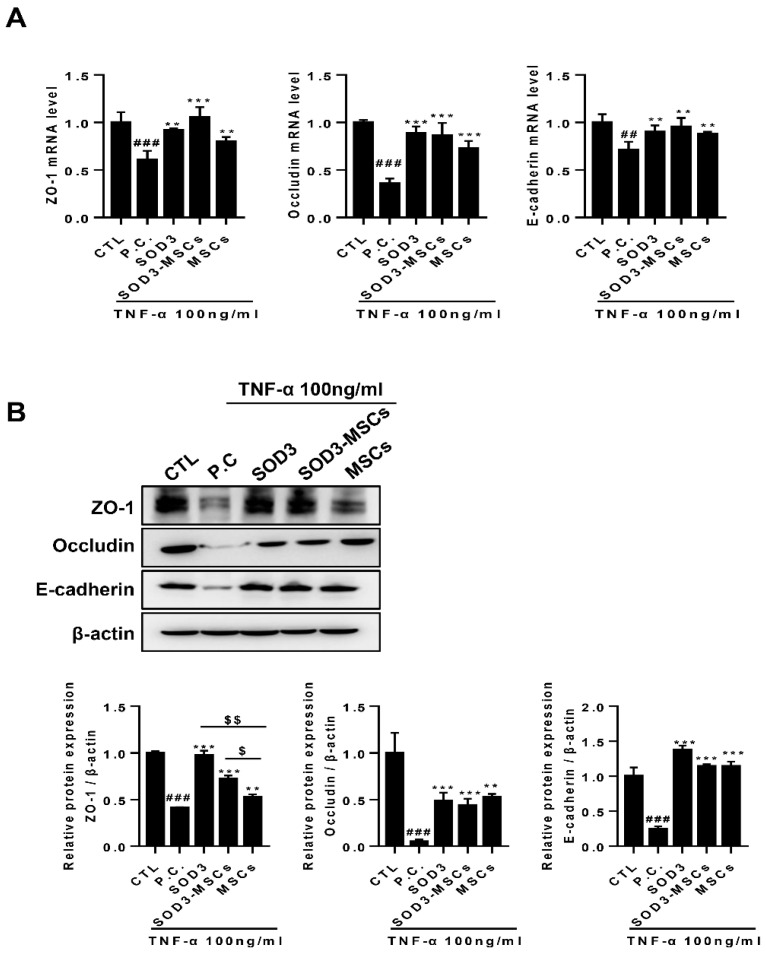
Protective effect of SOD3 in junctional damage of Caco-2 cells. (**A**) RNA was isolated from Caco-2 cells treated or co-cultured with SOD3 or SOD3-carrying MSCs, respectively, and the expression of tight/adherence junction markers (ZO-1, occluding and E-cadherin) was detected by quantitative PCR. (**B**) Proteins were harvested from cells of equal experimental settings and the expression of ZO-1, occludin, and E-cadherin was determined by western blotting. ## *p* < 0.01, ### *p* < 0.001 (CTL vs. P.C), ** *p* < 0.01, *** *p* < 0.001 (P.C vs. SOD3, SOD3-MSCs and MSCs), $ *p* < 0.05, $$ *p* < 0.01 (SOD3, SOD3-MSCs vs. MSCs). Results are shown as mean ± SD. Results show one representative experiment of at least three independent experiments.

To check the anti-inflammatory effects of SOD3, Caco-2 cells were treated with SOD3 or SOD3-MSCs. For SOD3 treatment, Caco-2 cells were pretreated with 300 units/mL of SOD3 for 3 h or co-cultured with SOD3-MSCs or naïve MSCs using transwell and then treated with 100 ng/mL of TNF-α for 24 h (Figure 1A). We selected this concentration based on our preliminary experiments and reports by other groups (Supplementary Figure S1A) [20,23]. Caco-2 cells were harvested 24 h after TNF-α treatment. MAPKs activation was assessed in harvested cells after determining the phosphorylation of ERK or JNK at various time points (Supplementary Figure S1B). TNF-α treatment activated the MAPK signaling in Caco-2 cells, represented by elevated phosphorylation of JNK and ERK. SOD3-MSCs co-culture significantly suppressed TNF-α–mediated p-JNK (Figure 2B). Moreover, both SOD3 treatment and SOD3-MSCs addition significantly down-regulated TNF-α–mediated p-ERK (Figure 1B). Furthermore, TNF-α treatment increased the expression of genes encoding various proinflammatory cytokines such as IL-6, IL-8, IL-1β, and IL-17C, involved in the progression of inflammatory bowel disease. Interestingly, SOD3 treatment by protein addition or SOD3-MSCs co-culture significantly suppressed the expression of proinflammatory genes compared to naïve MSC-treated group (Figure 1C). Although co-culture with MSCs significantly down-regulated the expression of proinflammatory genes in Caco-2 cells, SOD3 transduction further augmented the suppressive effect of MSCs (Figure 1C). In contrast, SOD3 increased the expression of a gene encoding the anti-inflammatory cytokine IL-10 (Figure 1C). In addition, SOD3 efficiently restored the increased level of ROS caused by TNF-α treatment (Supplementary Figure S1C).

### 2.2. SOD3 Maintains Intestinal Epithelial Barrier in Caco-2 Cells by Protecting the Disruption of Junctions

Given that pathogens can easily invade the submucosa and vasculature when intestinal mucosa permeability increases, which accelerates the inflammatory responses, we next sought to determine whether SOD3 can prevent TNF-α-mediated damage on junctions of the intestinal epithelium. Changes in the expression levels of ZO-1 and occludin, representative markers for tight junction, as well as E-cadherin, a marker for adherence junction, were assessed by qPCR and immunoblotting. The expressions of ZO-1, occludin, and E-cadherin at the mRNA level were significantly reduced by TNF-α (Figure 2A). It was observed that the expression levels of tight junction and adherence junction proteins were restored when treated with SOD3 or co-cultured with MSCs, and SOD3 transduction in MSCs restored the levels to a greater extent (Figure 2A). In particular, SOD3 or SOD3-MSCs restored the levels of ZO-1 and occludin to a similar extent to the basal level (Figure 2A). This protective effect of SOD3 against junction protein disruption was confirmed at the protein level and a similar pattern was observed (Figure 2B). In particular, SOD3 and SOD3-MSCs exhibited superior protective efficacy in the restoration of ZO-1 protein level compared to naïve MSCs (Figure 2B).

### 2.3. SOD3 and Sod3-MSCs Exert Protective Effects against DSS-Induced Murine Colitis

**Figure 3:**
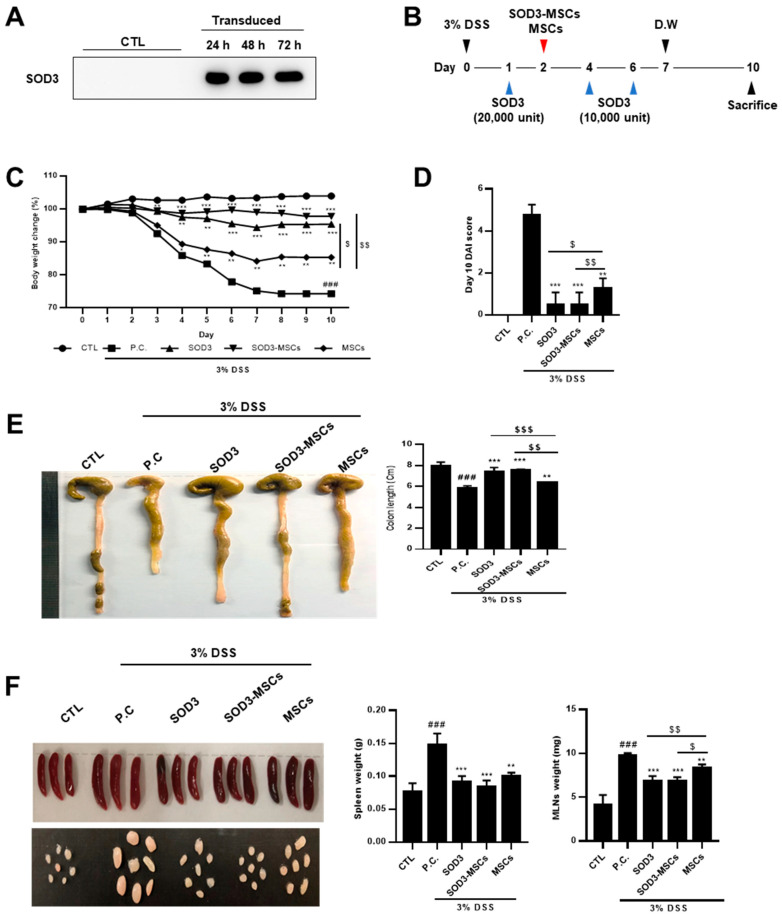
Administration of SOD3 protein and SOD3-MSC attenuate symptoms of murine colitis. (**A**) SOD3-transduced MSCs were confirmed for SOD3 secretion by performing western blotting using cell culture supernatant. (**B**) Timeline for in vivo colitis induction and SOD3 or SOD3-MSC administration. Five mice per group were used. SOD3 or SOD3-MSCs were i.p. injected on the designated day. (**C**) Body weight loss of each group was measured and shown as percentage loss. (**D**) Disease activity index for colitis severity on day 10 was scored. (**E**) The mice were sacrificed on day 10 and the length of colon was measured. (**F**) The weight of the spleen and MLNs was measured. D.W. = distilled water, ### *p* < 0.001 (CTL vs. P.C), * *p* < 0.05, ** *p* < 0.01, *** *p* < 0.001 (P.C vs. SOD3, SOD3-MSCs and MSCs), $ *p* < 0.05, $$ *p* < 0.01, $$$ *p* < 0.001 (SOD3, SOD3-MSCs vs. MSCs).

Before verifying the in vivo efficacy of SOD3-MSCs, SOD3 in the supernatant of transduced MSCs was measured by western blotting after cell culture for 24, 48, and 72 h. SOD3-transduced MSCs secreted significantly detectable levels of SOD3 in the culture supernatant (Figure 3A). Then, SOD3 protein or SOD3-MSCs were administrated into colitis mice to determine their therapeutic efficacy (Figure 3B). Upon colitis induction, the body weight of DSS-fed mice decreased, while that of SOD3 or SOD3-MSCs-injected mice were maintained without a significant loss (Figure 3C). More interestingly, SOD3 or SOD3-MSCs administration exhibited superior protective effects against colitis symptoms than the infusion of naïve MSCs (Figure 3C). On day 10, the disease activity index (DAI) was measured and MSCs injection significantly reduced DAI and SOD3-MSCs or SOD3 further decreased the index to a greater extent (Figure 3D). The colon length of the DSS-supplied mice was significantly reduced compared to the control group (Figure 3E). Remarkably, SOD3-treated mice and SOD3-MSCs-treated mice didn’t exhibit the shortening of colon length (Figure 3E). Given that the enlargement of the spleen and mesenteric lymph nodes (MLNs) reflects the extent of inflammation, the weight of spleen and MLNs increased significantly in DSS treated group compared to the control group (Figure 3F). In the SOD3 and SOD3-MSC-infused groups, significant restoration in the enlargement of spleen and MLNs was observed.

**Figure 4:**
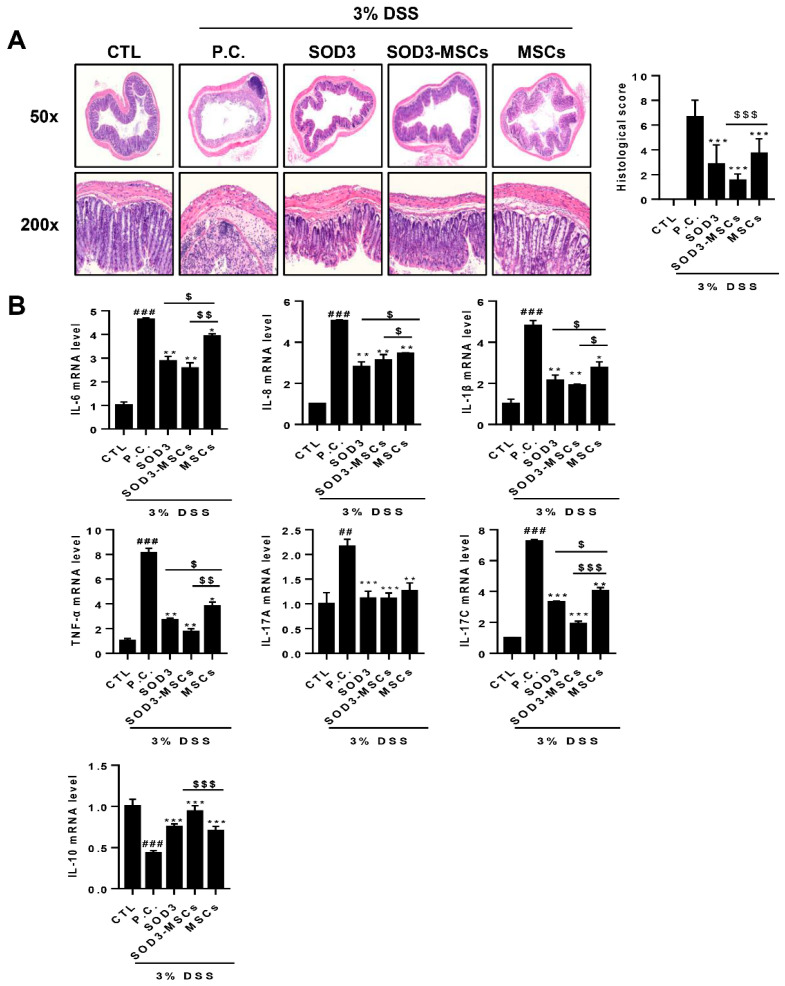
Histological and molecular analysis of colon samples from SOD3- or SOD3-MSC-treated mice. Colon sections were prepared and H&E staining was performed. (**A**) H&E stained sections were photographed and representative pictures are shown. Histopathological scores were measured by analyzing epithelial destruction and lymphocyte infiltration. (**B**) The expressions of pro- or anti-inflammatory genes in the colon were determined by real-time PCR. ## *p* < 0.01, ### *p* < 0.001 (CTL vs. P.C), * *p* < 0.05, ** *p* < 0.01, *** *p* < 0.001 (P.C vs. SOD3, SOD3-MSCs and MSCs), $ *p* < 0.05, $$ *p* < 0.01, $$$ *p* < 0.001 (SOD3, SOD3-MSCs vs. MSCs).

Upon histological examination, epithelial destruction accompanied by severe edema in submucosa and infiltration of lymphocytes was observed in the colon sections of DSS-treated mice. Both SOD3- and MSC-injected mice exhibited attenuated damage in their colons and SOD3-MSC infusion exerted a higher protective effects compared to the MSC-treated group (Figure 4A). In terms of the histological score, SOD3-MSCs showed the most potent protective effect against DSS-induced histological colonic damage. Consistent with the results observed in Caco-2 cells, we found that the expression of genes encoding proinflammatory cytokines, including IL-6, IL-8, IL-1β, TNF-α, IL-17A, and IL-17C, were significantly upregulated in the colons of DSS-treated mice (Figure 4B). MSC administration significantly downregulated the expression of these genes and more interestingly, SOD3 or SOD3-MSC treatment further suppressed the expression to greater extents, except for IL-17A (Figure 4B). Conversely, i.p. injection of SOD3 and SOD3-MSCs significantly upregulated the expression of IL-10 that was downregulated in the colons of DSS-treated mice (Figure 4B).

### 2.4. SOD3 Rescues DSS-Induced Activation of MAPK Signaling Pathway and Disruption of Epithelial Junction Barrier in Colon

**Figure 5:**
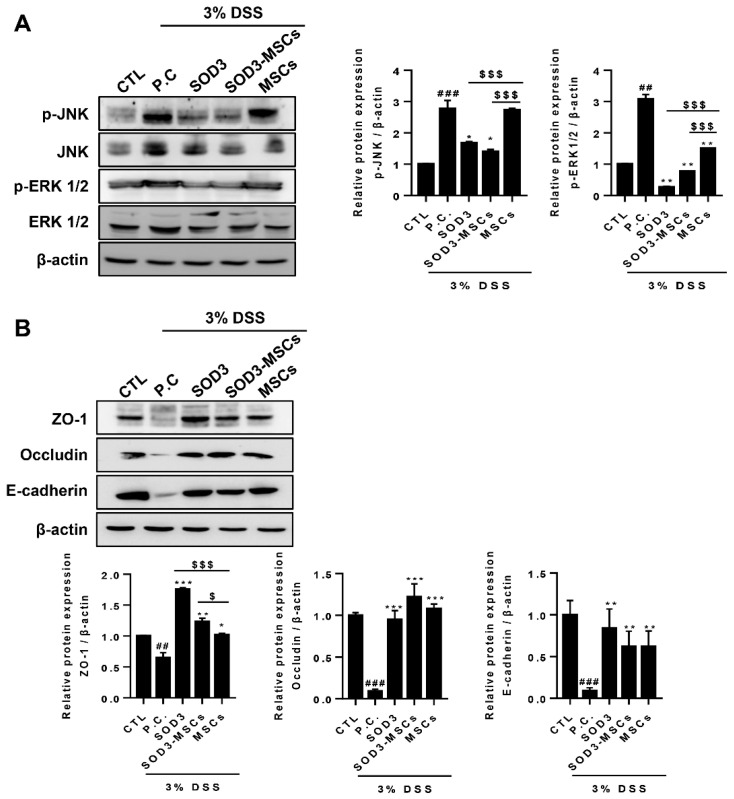
Expression of proteins in MAPK pathway and epithelial junctions in colons of colitis mice. (**A**) p-JNK, JNK, p-ERK1/2, and ERK1/2 were detected by immunoblotting. The expressions of p-JNK and p-ERK1/2 were quantified. (**B**) Expressions of ZO-1, occludin, and E-cadherin were analyzed by immunoblotting and quantified. ## *p* < 0.01, ### *p* < 0.001 (CTL vs. P.C), * *p* < 0.05, ** *p* < 0.01, *** *p* < 0.001 (P.C vs. SOD3, SOD3-MSCs and MSCs), $ *p* < 0.05, $$$ *p* < 0.001 (SOD3, SOD3-MSCs vs. MSCs). Results show one representative experiment of at least three independent experiments.

In our previous findings, TNF-α treatment activated the MAPK signaling pathway in Caco-2 cells, which in turn affects the junctional stability of the intestinal epithelium. Therefore, we next sought to confirm the disruption of the intestinal barrier in the colons of DSS-treated mice, and its protection by the administration of SOD3 or SOD3-MSCs. DSS-induced colitis mice showed increased phosphorylation of JNK and ERK 1/2 and both SOD3 and SOD3-MSCs significantly downregulated the phosphorylation (Figure 5A). Particularly, SOD3 protein injection exhibited the most potent suppressive effects on ERK phosphorylation (Figure 5A). Changes in the expression levels of tight junction and adherence junction proteins were assessed. Consistent with in vitro findings, SOD3 and SOD3-MSCs efficiently protected against the DSS-induced loss of ZO-1, occludin, and E-cadherin proteins (Figure 5B). Interestingly, the administration of SOD3 protein significantly elevated the level of ZO-1 protein to an extent greater than the basal expression observed in the control group (Figure 5B).

### 2.5. SOD3 Protein and SOD3-MSCs Prevent Inflammatory Damage in Intestinal Organoids

**Figure 6:**
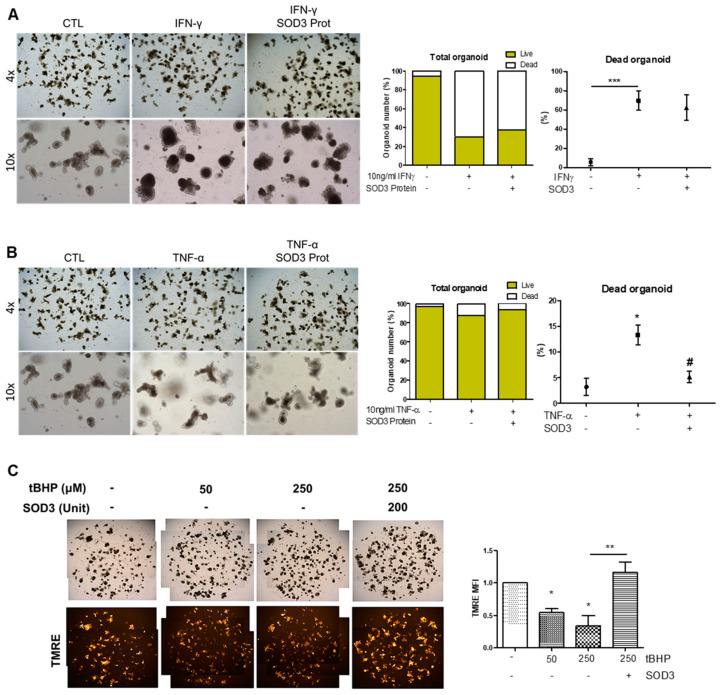
The protective action of SOD3 protein on IOs against pro-inflammatory and oxidative stress. (**A**,**B**) The representative bright-field images and live/dead assessment of IFN-γ (**A**) or TNF-α (**B**) treated IOs on culture day 5. Cytokines were added on culture day 3. (**C**) To induce the generation of reactive oxygen species, tBHP was added to IO culture then their mitochondrial activity was determined by TMRE staining. Mean fluorescence intensity (MFI) of CTL was standardized as 1. At least three individual experiments were performed. * *p* < 0.05, ** *p* < 0.01, *** *p* < 0.001 (CTL vs. IFN-γ and TNF-α in **A**,**B**; CTL vs. others in **C**), # *p* > 0.05 (TNF-α vs. SOD3 protein). Results are shown as mean ± SD. Results show one representative experiment of at least three independent experiments.

Considering that SOD3 or SOD3-overexpressed MSCs can efficiently reduce the epithelial damage in the intestine both in vivo and in vitro, we utilized an organoid culture system to evaluate the impact of SOD3 on epithelial homeostasis and injury response. We confirmed that no visible effect on organoid growth morphology (Supplementary Figure S2A), nor signature gene expression pattern (Supplementary Figure S2B), was observed upon SOD3 protein treatment. On the contrary, when IFN-γ or TNF-α were treated on culture day 3 for 48 h to induce damage in intestinal organoids (IOs), organoid growth and maturation were significantly impeded, accompanied by a loss of budding (Figure 6A,B). Although the co-administration of SOD3 protein on IFN-γ-treated IOs could not reverse the injury response of the intestinal epithelium, interestingly, the increased proportion of dead organoids in the TNF-α-treated group was significantly reduced in the presence of SOD3 protein (Figure 6A,B). Since SOD3 is one of the representative regulators of ROS, we investigated the beneficial impact of SOD3 protein on IOs in the presence of oxidative stress. For this purpose, we treated the oxidative damage inducer, tBHP to IOs for 2 h then conducted TMRE staining to evaluate the level of mitochondrial activity. We found a dose-dependent reduction in tetramethylrhodamine, ethyl ester (TMRE) intensity upon tBHP treatment, whereas SOD3 protein could prevent ROS-mediated mitochondrial damage in IOs. These data indicate that SOD3 protein can provide specific protections against a proinflammatory cytokine, TNF-α, as well as oxidative stress-mediated mitochondrial dysfunction in the intestinal epithelium.

**Figure 7:**
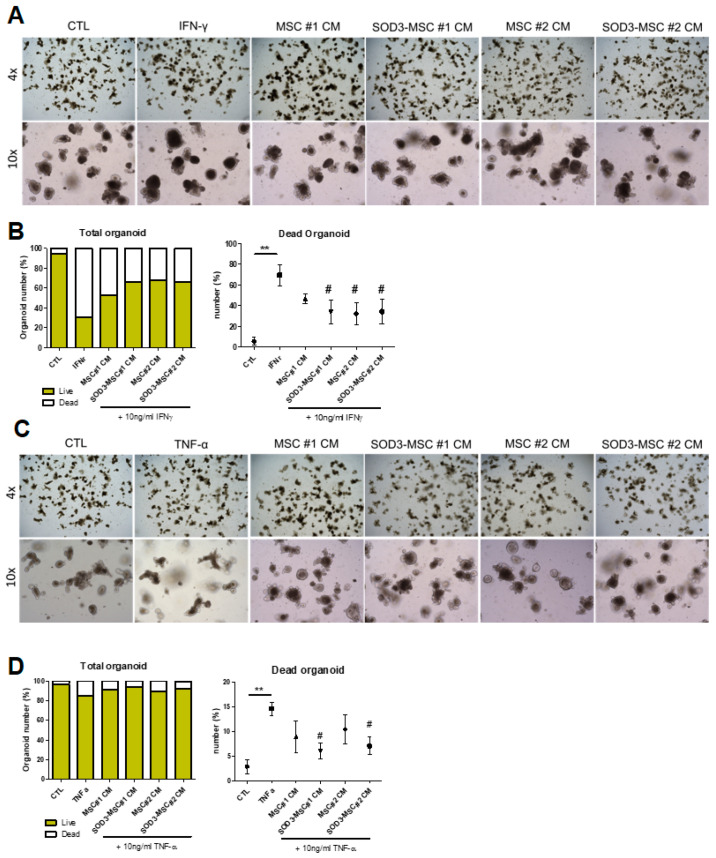
The protective impact of SOD-MSCs on pro-inflammatory cytokine-treated IOs. (**A**,**C**) The representative bright-field images of IFN-γ (**A**) or TNF-α (**C**) treated IOs. Upon IFN-γ treatment, IOs lost their budding structure and unorganized cellular aggregates appeared instead due to a broken epithelial lining. (**B**,**D**) Live/dead organoid counting results upon IFN-γ (**B**) or TNF-α (**D**) treatment. Both control- and SOD3-MSC-CM protected IOs from pro-inflammatory damage. At least three individual experiments were performed. ** *p* < 0.01 (CTL vs. IFN-γ or TNF-α), # *p* < 0.05 (IFN-γ or TNF-α vs. MSC-CM). Results are shown as mean ± SD. Results show one representative experiment of at least three independent experiments.

Given that IOs are made up of multiple cells and are sensitive to various paracrine factors, we separately evaluated the effects of paracrine factors derived from MSC or SOD3-MSCs on IO composition or damage responses. First, IOs were cultured with conditioned media (CM) from control- and SOD3-MSCs for 5 days, and their organoid formation efficiency was evaluated by budding number counting. No significant difference in the morphology was observed between control- and MSC-CM treated groups (Supplementary Figure S2A). We then screened several marker genes of IOs for intestinal epithelial cells (IECs) and found that MSC-CM can constantly induce an absorptive, enterocyte differentiation (Apoa4-expressing cells) compared to control. In addition, among the secretory cell lineages, Paneth cells (Lysozyme^+^) and goblet cells (Tff^+^) were increased in the MSC-CM cultured group (Supplementary Figure S3B). In particular, goblet cell differentiation was significantly upregulated in SOD3-overexpressed CM than in control CM (Supplementary Figure S3B). We next determined whether MSC-CM can have any beneficial effects on the intestinal epithelial injuries. Co-culture of MSC-derived CM could prevent IFN-γ-induced organoid death (Figure 7A,B). More interestingly, only CMs obtained from SOD3-MSCs, not from control MSCs, provided significant protection on TNF-α-mediated IO damage (Figure 7C,D). These results collectively imply that SOD3 protein or SOD3 delivery by MSCs can be beneficial for the prevention of epithelial damage in enteritis, and that paracrine factors from MSCs might have different protective mechanisms against intestinal epithelial damage.

## 3. Discussion

A secretory extracellular enzyme, SOD3 is one of the most important antioxidants in mammals [24]. We have previously shown its therapeutic potential in many inflammatory diseases; however, since the half-life of SOD3 is short, it is limited in its use as a therapeutic substance by itself. Fortunately, it has been reported that the use of SOD3-MSCs infected with Lenti-SOD3 in MSCs may play an effective anti-inflammatory role compared to the use of SOD3 or MSCs alone [16,25]. In this study, we suggest SOD3-MSCs as a new therapeutic agent targeting intestinal epithelial recovery in the experimental colitis model.

The intestinal epithelium acts as a selective barrier to the outside environment and the damage to the mucosal barrier increases intestinal permeability, promotes intestinal cell exposure to the toxic lumen contents, and thus promotes intestinal inflammation. Therefore, the gut mucosal barrier is the most important protective membrane for maintaining intestinal immune tolerance [26]. Given that inflammatory mediators such as TNF-α and IFN-γ can disrupt the barrier integrity, and that targeting TNF-α can restore the barrier function [23], we first investigated the cytotoxic effect of TNF-α on the intestinal epithelium using human IEC Caco-2 cells. In addition, we found that tight junction protein levels of intestinal epithelium were reduced by TNF-α [22], and SOD3, as well as SOD3-MSCs, can restore this phenomenon. Also, TNF-α activated the MAPKs pathway in Caco-2 cells as previously observed [3]. Since the production of proinflammatory cytokines can be further increased by the activated MAPKs pathway [3,27], our results show that SOD3 and SOD3-MSCs effectively inhibited the activation of p-JNK and p-ERK1/2. This implies the therapeutic effect of our method in modulating the TNF-α mediated inflammation.

Next, we confirmed the in vivo impact of SOD3, SOD3-MSCs, and MSCs in a DSS-induced colitis model. Consistent with the results shown in this study, the treatment of MSC itself has been demonstrated to exert protective or therapeutic efficacy against various immune disorders including IBD through the production of immunoregulatory paracrine factors including prostaglandin E_2_, indoleamine 2,3-dioxygenase-1, and transforming growth factor β1 [28,29]. In the present study, we tried to assess the improved effect of SOD3-introduced MSCs compared to the basal effect of MSC administration itself. DSS is known to cause intestinal inflammation by damaging the lining of the epithelial monolayer of the intestine, allowing proinflammatory intestinal contents to propagate to the underlying tissue [30]. It was noted that the weight loss and DAI showed the least changes in the SOD3 and SOD3-MSCs groups. Also, we further confirmed that excessive MAPKs activation and increased intestinal permeability were reduced by the treatment with SOD3 or SOD3-MSCs in the DSS-induced colitis model as observed in vitro. In particular, the SOD3-MSCs-treated group showed the most significant recovery from the disease compared to others in terms of histological assessment and biochemical analysis, implying the prominent contribution of SOD3 action in these phenomena. Considering that the integrity of the tight junction is related to the severity of IBD [26,31], we suggest that SOD3-MSCs can ameliorate the IBD-associated intestinal damage to the epithelium via remodeling the tight junction, as this might be dependent on their superior ROS-regulating capacity than control MSCs. One can envision that the improved therapeutic function of SOD3-MSCs might result from the combination of SOD3 with paracrine factors from MSCs, as well as the improved delivery of SOD3 to inflammatory sites due to the migratory abilities of MSCs toward sites where inflammation is excessive. During the inflammation, not only immune cells but also IECs are stimulated by proinflammatory cytokines to produce ROS [32]. ROS is removed by scavengers in homeostatic conditions; however, if excessive inflammation or deficient intestinal barrier without regeneration persists, ROS accumulates and further aggravates IEC damage [33,34]. Importantly, elevated ROS levels have been reported in IBD-affected patients with decreased SOD3 in IEC [35,36], while the expression level of other isoforms remained unchanged (in case of SOD1) or even increased (in case of SOD2) [19]. Moreover, the total SOD activity seems to exhibit a positive correlation pattern with disease severity, and it is upregulated in the activated state and downregulated in remission [37,38]. Collectively, SODs modulate ROS levels in the intestine and dysregulated SOD expression leads to the aggravation of IBD symptoms. Here we suggest that an increase in SOD3 activity can improve the intestinal epithelial damage both in vitro and in vivo. Interestingly, a recent work has shown that SOD1 knockout mice exhibited increased susceptibility for DSS-induced colitis with upregulation of ROS and p38-MAPK signaling, in which oral administration of SOD could ameliorate the disease phenotype [39]. Considering the specific role and expression pattern of SOD isoforms in the intestine, it would be interesting to evaluate the therapeutic potential of SOD2- or SOD3 treatment in this model.

Recently, the organoid system has been widely applied from basic research to pre-clinical studies. To study how SOD3-MSCs can rescue the impaired intestinal epithelium in the context of IBD, we prepared IOs from mice and treated typical proinflammatory cytokines IFN-γ and TNF-α, since these cytokines drive the inflammation-mediated loss of IECs, leading to an IBD-like phenotype [40]. Injection of recombinant TNF-α or TNF-α expressing plasmid was reported to accelerate epithelial cell turnover and activates apoptotic death by upregulating TNF receptors in villus tip-residing cells [41]. Persistent exposure of IFN-γ to IEC line T84 induces Wnt pathway antagonist DKK1 production and inhibits cell proliferation [42]. Also, both cytokines can impair the intestinal barrier function and increase permeability by decreasing the tight junction proteins as well as inducing cytoskeletal reorganization [43,44]. Notably, some inflammatory features are conserved in patient-derived IOs [45,46] for several passages and long-term treatment of inflammatory cytokines can elicit IBD-related immunophenotype in human and mouse IOs [45,47]. Therefore, the administration of IFN-γ and TNF-α to IOs can mimic the nature of intestinal damage in IBD and is useful for proving the therapeutic potential of MSC-CMs. Here we found that both control- and SOD3-MSC-derived CM can reduce IFN-γ -associated IEC loss, while CM from SOD3-MSCs provides superior prevention against TNF-α induced death than that from normal MSCs. Given that the SOD3 protein itself exerts specific protection against TNF-α but not IFN-γ in IOs, it would be interesting to evaluate the benefits of control- and SOD3-MSCs against various IBD-mimicking combinations such as IL1-β and flagellin, and to explore the therapeutic factors responsible for each stimulus. In addition, we reported the anti-oxidative effect of SOD3 protein on tBHP-treated IOs. Regulating ROS level in IECs is important for maintaining the integrity of the intestinal structure, and ROS-accumulation in damaged cells must be removed via mitophagy or autophagy prior to the repair process [48,49,50]. Therefore, the enhanced anti-oxidative capacity of SOD3-MSCs would upregulate the therapeutic outcome of MSCs application in IBD by potentiating the endogenous regeneration in the intestinal epithelium. The limitation of the present study is that the concentration of SOD3 in the cells or the media was not quantified, and therefore are not comparable with the concentration of purified SOD3 protein. Further investigations are required to quantitatively compare the concentration of SOD3 from transduced MSCs with that of purified SOD3 protein and to explain the dose-dependent consequences of SOD3 on the cell signaling. Taken together, our findings demonstrate that SOD3 protein or SOD3-MSCs might be promising therapeutics for the treatment of IBD through the downregulation of inflammatory responses, as well as the maintenance of epithelial junctional integrity.

## 4. Materials and Methods

### 4.1. Preparation of SOD3 and SOD3-MSCs

SOD3 was prepared as previously described [14]. Briefly, HEK 293 cells were transiently transfected with the SOD3 plasmid construct for 48 h. The supernatant was collected and purified using a Ni Sepharose 6 fast flow bead (11-0012-38, GE Healthcare, Chicago, IL, USA) and then the purified SOD3 activity was measured and calculated using a SOD assay kit (S311, Dojindo Molecular Technologies, Kumamoto, Japan) immediately before the experiment. For the treatment of SOD3 in mice or cells, SOD3 was filtered to eliminate endotoxins using a syringe filter (0.22 μm pore size, BIOFIL, Barcelona, Spain).

SOD3-MSCs were generated by infecting MSCs with the Lenti-SOD3 virus as previously described [51]. Briefly, human MSCs from umbilical cord blood were provided from Kangstem Biotech (Seoul, Korea). MSCs were prepared according to previously described methods [52], cultured in KSB-3 culture medium (Kangstem Biotech), and supplemented with 10% FBS (fetal bovine serum, GenDEPOT, Hanam, Korea) and 1% penicillin-streptomycin (LS202–02, Welgene, Gyeongsan-si, Korea) at 37 °C, 5% CO_2_. Lenti-M1.9-SOD3 was provided by Chungnam National University and used to infect MSCs. In our routine preparation, the titer was approximately 1.0 × 10^7^ TU/mL, and MSCs were transduced at 5 multiplicity of infection (MOI) concentration. After incubating MSCs for 24 h, the SOD3 lentiviral vector was treated with MSCs. To confirm the overexpression of SOD3 in MSCs, the culture medium was collected every 24 h after infection and SOD3 expression was determined by western blotting.

### 4.2. Caco-2 Cell Culture and TNF-α Treatment

The human colon adenocarcinoma cell line, Caco-2 cells were provided by Dong-A University, Busan, Korea and cultured in DMEM (GenDEPOT) supplemented with 10% FBS (GenDEPOT) and 1% penicillin-streptomycin (Welgene), and 1% non -essential amino acid (M7145, Sigma-Aldrich, Saint Louis, MO, USA) at 37 °C, 5% CO_2_. Caco-2 cells were pretreated with 300 unit/mL SOD3 for 3 h after seeding in 6 well plates (Costar, 3516, NY, USA). Subsequently, recombinant human TNF-α (100 ng/mL concentration, 210-TA, R&D system, MN, USA) was added to the cell culture medium.

### 4.3. Caco-2 Cells and MSCs Co-Culture Assay

Co-culture was performed using transwell plates (0.4 μL pore size; Costar). Caco-2 cells were seeded in the lower chambers of the plates, and MSCs or SOD3-MSCs were seeded in the upper chambers. The cells were plated at a 1:1 ratio (Caco-2: MSCs or Caco-2: SOD3-MSCs). Caco-2 cells were seeded at 2 × 10^6^ cells/well and after 3 h, MSCs or SOD3-MSCs was plated. All cells were seeded and cultured in Caco-2 growth medium. After 24 h, the Caco-2 cells were stimulated with TNF-α.

### 4.4. RNA Extraction for Quantitative PCR from Caco-2 and Colitis Mice Colon

Total RNA was extracted using the TRIzol reagent (Invitrogen, Carlsbad, CA, USA) following the manufacturer’s instructions. qPCR was performed using a Light Cycler 96 (Roche, Basel, Switzerland) and a QuantiTech SYBR Green PCR kit (Qiagen, Hilden, Germany). Previously designed primers for ZO-1, Occludin, E-cadherin, IL-6, IL-8, IL-10, IL-1β, IL-17A, IL-17C, and GAPDH are listed in Table 1 (human) and Table 2 (mouse). Relative expression levels of each marker were normalized to the CT value of GAPDH.

### 4.5. Western Blotting

Caco-2 cells or colitis mice’s colons were harvested and lysed with RIPA buffer (Thermo Fisher Scientific, Waltham, MA, USA) and 1 mM PMSF, protease inhibitor cocktail. Protein concentration was measured and equal amounts of proteins were loaded and separated on SDS-PAGE (EBA-1041, Elpis Biotech, Korea), and transferred to a polyvinylidene difluoride membrane (PVDF, IPVH00010, Sigma Aldrich). The membranes were incubated with antibodies for ZO-1 (Abcam, Cambridge, UK), Occludin, β-actin (Santa Cruz Biotechnology, Dallas, TX, USA), E-cadherin, p-P38, p38, p-ERK1/2, and ERK1/2 (Cell Signaling Technology, Danvers, MA, USA). Proteins were detected by enhanced chemiluminescence (K-12045-D-50, Advansta, San Jose, CA, USA) and visualized using the LAS 3000 detection system (Fujifilm Corporation, Tokyo, Japan).

### 4.6. Mice and Colitis Induction

8-week-old male C57BL/6 mice were purchased from Central Laboratory Animal, Inc., Seoul, Korea, and kept under specific pathogen-free conditions. All procedures involving animal research were conducted in accordance with the Laboratory Animals Welfare Act, the guide for the care and use of laboratory animals, and the guidelines and policies for rodent experiment provided by the IACUC (Institutional Animal Care and Use Committee) in the school of medicine, the Catholic University of Korea (Approval number: CUMS-2019-0285-05). Colitis was induced by administering 3% (*w*/*v*) DSS in drinking water for 7 days. Negative control mice received D.W (distilled water) only. Colitis severity was measured by evaluating the disease activity index (DAI) through the scoring of weight loss, diarrhea, blood excrement, bleeding, and coat roughness (grade from 0–5 on the severity of each index). SOD3, SOD3-MSCs, and MSCs were prepared by the same procedure used for in vitro experiments. After purification, SOD3 was concentrated so that the final volume per mouse did not exceed 200 μL, and then filtered. The activity was calculated so that the final concentration of each mouse was 40,000 units and was injected over the course of three intraperitoneal injections. SOD3-MSCs and MSCs were harvested immediately prior to injection. Harvested cells were amounted to 2 × 10^6^ cells/mouse, and were dissolved in 200 μL of 1× PBS and administrated intraperitoneally on day 2.

### 4.7. Tissue Harvesting

On day 10, mice were sacrificed and gastrointestinal tract was harvested. The mesenteric lymph nodes and spleens were collected, their weights were measured, and the lengths of the colons were measured. The colons were then washed with cold 1× PBS and prepared for H&E staining and PCR or western blot analysis. Samples for H&E staining were fixed in a 4% PFA (paraformaldehyde solution; T&I, BPP-9004, Seoul, Korea) solution for 24 h, and then a paraffin block was prepared. Samples used for PCR or western blot were stored in a LN2 tank and RNA and protein were extracted.

### 4.8. Histopathological Evaluation

5 μm-thick sections from paraffin blocks were prepared and stained with H&E. Leukocyte infiltration and intestinal damage were graded blindly. The epithelial destruction was scored by determining destructive proportion of entire epithelium and submucosal edema (graded as 0–4) and the infiltration of lymphocytes into the lamina propria and submucosa (graded as 0–4). These were blindly graded.

### 4.9. Intestinal Organoids Culture

To generate intestinal organoids (IOs), intestinal crypts were isolated from the mouse small intestine as previously described [53,54]. In brief, the harvested mouse small intestine was longitudinally opened and villi were removed by gentle scraping with a glass coverslip. The tissue was cut into small pieces (2–3 mm) then incubated in gentle cell dissociation solution (Stemcell Technologies, Vancouver, BC, Canada) for 20 min at room temperature followed by vigorous shaking to dislocate the crypts. After the removal of the intestinal segment, the supernatant containing crypts was washed with PBS at 400× *g* for 5 min at 4 °C to pellet the crypts. A total of 300 crypts mixed with 25 μL Matrigel (at 1:1 ratio) were seeded onto one well of 24 well plates. Organoids were kept in growth media composed of DMEM/F-12 (Gibco, Grand Island, NY, USA), N2 (Gibco), B27 (Gibco), 1 mM N-acetylcysteine (Sigma-Aldrich), 100 ng/mL Noggin (Peprotech, Rocky Hill, NJ, USA), 100 ng/mL murine EGF (Peprotech) and 10% of R-spondin conditioned media. Media change was done every 2–3 days and the subculture of organoids was conducted every 5–7 days.

### 4.10. Damage Induction in Intestinal Organoids

On culture day 3, proinflammatory cytokines IFN-γ and TNF-α were treated with intestinal organoids for 48 h then morphological change was assessed based on the budding structure and membrane integrity. Morphologically, healthy organoids have a clear epithelial lining and contain several buddings with a dense lumen due to normal epithelial cell turnover, while dead organoids are characterized by a broken epithelial layer and cellular aggregates. To introduce oxidative stress into the organoid culture system, tert-Butyl hydroperoxide (tBHP) (Sigma-Aldrich) was treated with organoids on culture day 3 for 2 h, then mitochondrial activity was determined using Tetramethylrhodamine ethyl ester (TMRE) staining as per manufacturer’s instruction. The image of organoids was captured using EVOS FL Auto 2 (Thermo Fisher Scientific) and fluorescent intensity was analyzed with Image J software (National Institutes of Health, Bethesda, MD, USA).

### 4.11. Collection of MSC-Conditioned Media (CM) for IOs Culture

To generate MSC-CM for the IOs culture, 5 × 10^5^ cells of control- and SOD3-MSCs were seeded on a 100 mm culture dish and supplemented with KSB-3 media containing 10% FBS. The next day, the media was discarded and cells were washed with PBS three times, then 8 mL of DMEM/F-F12 media was added. 24 h later, the media was collected and centrifuged at 400× *g* for 5 min to remove cell debris, then supplement factors required for the organoid growth as described in Section 4.9 were added. Final CM was stored at −80 °C until use.

### 4.12. RNA Isolation from Organoids and Quantitative PCR

Organoid RNA extraction was performed using RNeasy Mini Kit (Qiagen). PCR was performed using SYBR Green reagent (Thermo Fisher Scientific) on QuantStudio 1 Real-Time PCR System (Thermo Fisher Scientific). Used primer sequences are provided in Table 3. Relative expression levels of each marker were normalized to the CT value of GAPDH.

## Figures and Tables

**Table 1:** Human primer sequence for qPCR.

Gene	Forward Primer	Reverse Primer
IL-6	CACTCACCTCTTCAGAACGA	CTGTTCTGGAGGTACTCTAGG
IL-8	TGGCTCTCTTGGCAGCCTTC	TGCACCCAGTTTTCCTTGG
IL-10	TTACCTGGAGGAGGTGATGC	GGCCTTGCTCTTGTTTTCAC
IL-1β	CCACAGACCTTCCAGGAGAATG	GTGCAGTTCAGTGATCGTACAGG
IL-17C	GCCCTCAGCTACGACCCAGTG	AGCTTCTGTGGATAGCGGTCCT
ZO-1	GTCCAGAATCTCGGAAAAGTGCC	CTTTCAGCGCACCATACCAACC
Occludin	ATGGCAAAGTGAATGACAAGCGG	CTGTAACGAGGCTGCCTGAAGT
E-cadherin	GCCTCCTGAAAAGAGAGTGGAAG	TGGCAGTGTCTCTCCAAATCCG
GAPDH	GTCTCCTCTGACTTCAACAGCG	ACCACCCTGTTGCTGTAGCCAA

**Table 2:** Mouse primer sequence for qPCR.

Gene	Forward Primer	Reverse Primer
IL-6	AGACAGCCACTCACCTCTTCAG	TTCTGCCAGTGCCTCTTTGCTG
IL-8	CTCTATTCTGCCAGATGCTGTCC	ACAAGGCTCAGCAGAGTCACCA
IL-10	CGGGAAGACAATAACTGCACCC	CGGTTAGCAGTATGTTGTCCAGC
IL-1β	TGGACCTTCCAGGATGAGGACA	GTTCATCTCGGAGCCTGTAGTG
IL-17C	GTTGCCTACTGGGATGACCC	ACCTGGCACTTCGAGTTAGC
IL-17A	CAGACTACCTCAACCGTTCCAC	TCCAGCTTTCCCTCCGCATTGA
TNF-α	GGTGCCTATGTCTCAGCCTCTT	GCCATAGAACTGATGAGAGGGAG
ZO-1	GTTGGTACGGTGCCCTGAAAGA	GCTGACAGGTAGGACAGACGAT
Occludin	TGGCAAGCGATCATACCCAGAG	CTGCCTGAAGTCATCCACACTC
E-cadherin	GGTCATCAGTGTGCTCACCTCT	GCTGTTGTGCTCAAGCCTTCAC
GAPDH	CATCACTGCCACCCAGAAGACTG	ATGCCAGTGAGCTTCCCGTTCAG

**Table 3:** Organoid primer sequence for qPCR.

Gene	Forward	Reverse
Lgr5	GGGAGCGTTCACGGGCCTTC	GGTTGGCATCTAGGCGCAGGG
Tff3	TAATGCTGTTGGTGGTCCTG	CAGCCACGGTTGTTACACTG
Apoa4	GCCCAGTGAGGAGCCCAGGA	CCACATTGGCCACCTGGTCCG
Neurog3	GCATGCACAACCTCAACTC	TTTGTAAGTTTGGCGTCATC
GAPDH	CATCACTGCCACCCAGAAGACTG	ATGCCAGTGAGCTTCCCGTTCAG
Lysozyme	TGAACGTTGTGAGTTTGCCA	TGAGCTAAACACACCCAGTCG
Ki67	CTGCCTGCGAAGAGAGCATC	AGCTCCACTTCGCCTTTTGG

## Data Availability

The data presented in this study are available on request from the corresponding author.

## Supplementary Materials

The following are available online at https://www.mdpi.com/article/10.3390/ijms22126431/s1, Figure S1: TNF-α toxicity evaluation on Caco-2 cells, Figure S2: Assessment of morphology and marker gene expression of IOs cultured with SOD3 protein, Figure S3: Assessment of morphology and marker gene expression of IOs upon co-culture with MSC-CM.
