# Supplementary material for: Superoxide Dismutase 3-Transduced Mesenchymal Stem Cells Preserve Epithelial Tight Junction Barrier in Murine Colitis and Attenuate Inflammatory Damage in Epithelial Organoids
Source: Int J Mol Sci. 2021 Jun 16;22(12):6431. doi: 10.3390/ijms22126431 (PMC8233984; doi:10.3390/ijms22126431)
Supplement: Supplementary file 1 [file ijms-22-06431-s001.zip › ijms-1246276-supplementary.pdf]

Supplementary Figures

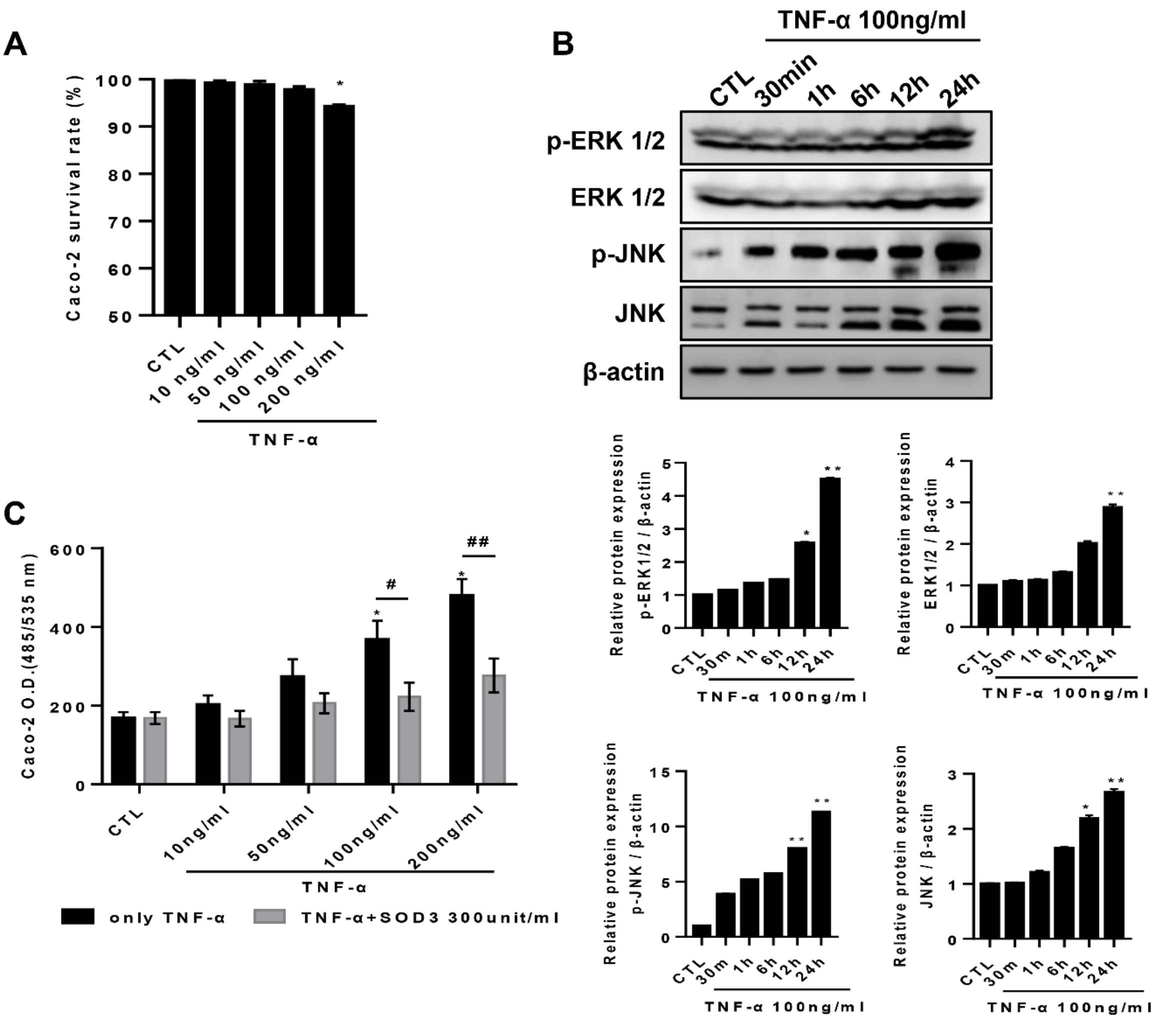

Figure S1. TNF-α toxicity evaluation on Caco-2 cells. (A) The survival rate of caco-2 was measured by MTT assay. (B) p-JNK, JNK, p-ERK1/2 and ERK1/2 were detected by immunoblotting. The expressions of p-JNK, JNK, p-ERK1/2 and ERK1/2 were quantified. (C) Inhibitory effect of SOD3 on TNF-α mediated ROS level was determined by measuring DCF-DA. CTL: untreated control; * $p < 0.05$, ** $p < 0.01$, *** $p < 0.001$ (CTL vs. TNF-α treated), #$p < 0.05$, ##$p < 0.01$ (only TNF-α vs. TNF-α+SOD3 300 unit/ml). Results are shown as mean ± SD. All data represent three independent experiments.

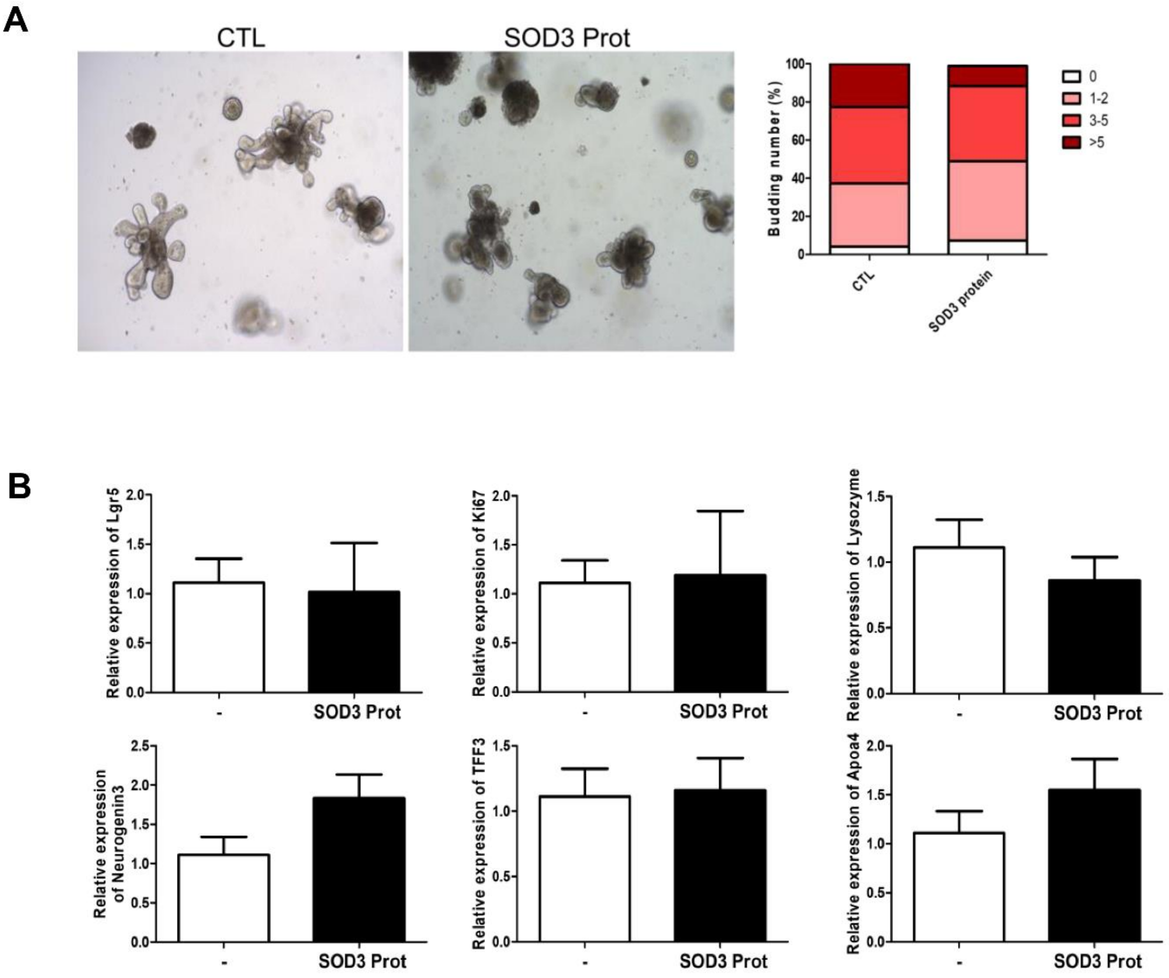

Figure S2. Assessment of morphology and marker gene expression of IOs cultured with SOD3 protein. (A) The representative bright-field images of control- and SOD3 (200 unit). SOD3 treated IOs on culture day 5 and budding count results are shown. (B) The quantitative real-time PCR analysis for representative markers of intestinal epithelial cells including Lgr5 (intestinal stem cells), Ki67 (proliferative cells), lysozyme (Paneth cells), Neurog3 (enteroendocrine cells), Tff3 (Goblet cells) and Apoa4 (enterocytes) in control- and SOD3-treated IOs. At least three individual experiments were performed. Data are presented as mean ±SD.

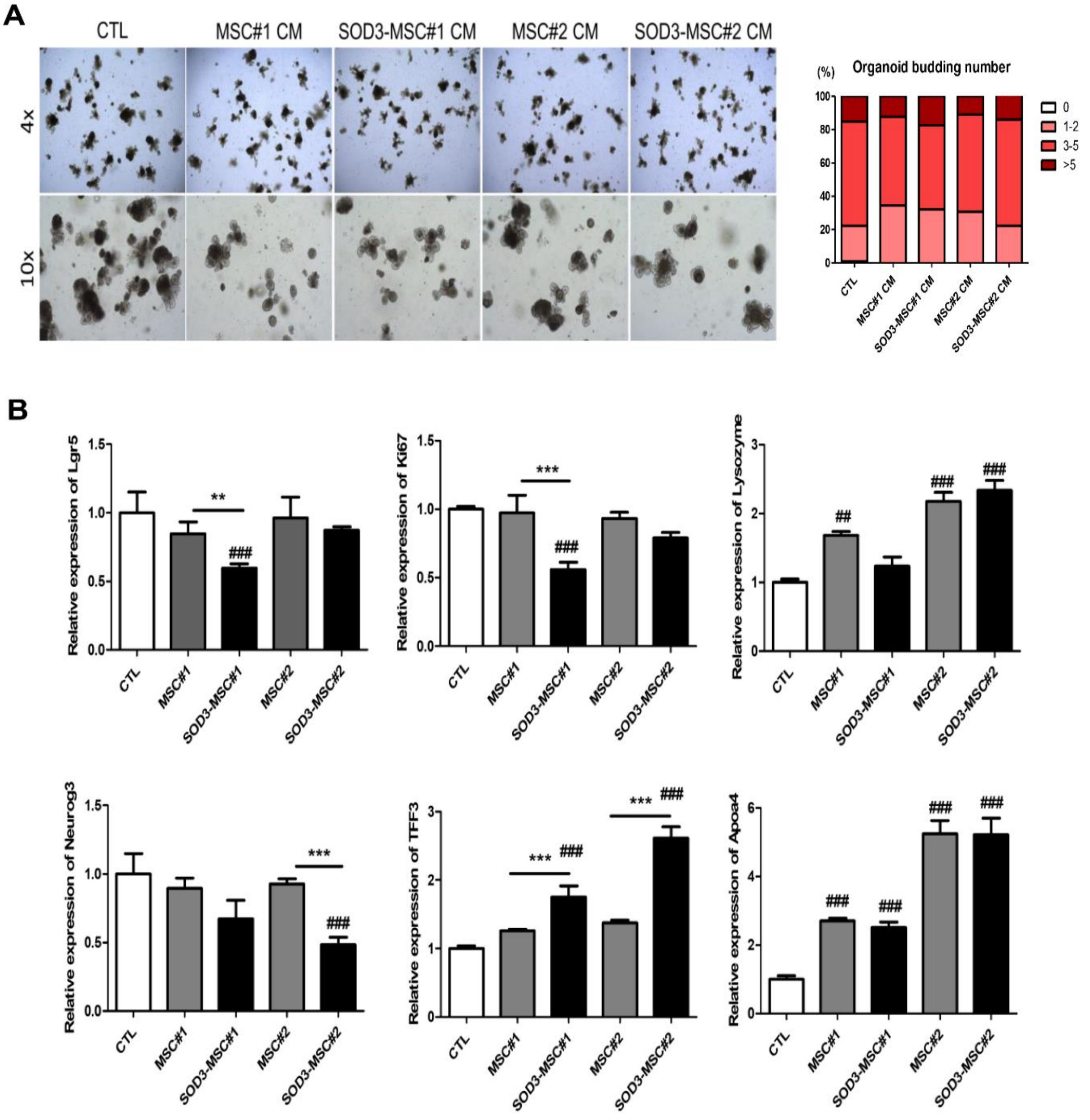

Figure S3. Assessment of morphology and marker gene expression of IOs upon co-culture with MSC-CM. (A) The representative bright-field images of IOs on culture day 5 and budding count results are shown. (B) The quantitative real-time PCR analysis for representative markers of intestinal epithelial cells. At least three individual experiments were performed. Data are presented as mean ±SD. ## $p < 0.01$, ### $p < 0.001$ (CTL vs. MSCs), ** $p < 0.01$, *** $p < 0.001$ (MSCs vs. SOD3-MSCs)
